# Measuring health system responsiveness at facility level in Ethiopia: performance, correlates and implications

**DOI:** 10.1186/s12913-017-2224-1

**Published:** 2017-04-11

**Authors:** Bereket Yakob, Busisiwe Purity Ncama

**Affiliations:** 1 Descpline of Public Health Medicine, School of Nursing & Public Health, University of KwaZulu–Natal, Durban, South Africa; 2 Health Economics and HIV/AIDS Research Division (HEARD), University of KwaZulu–Natal, Durban, South Africa

**Keywords:** Responsiveness, HIV/AIDS, Treatment and care services, Perceived quality of care, Wolaita Zone, Ethiopia, Satisfaction with services, Financial fairness, Traditional medicine

## Abstract

**Background:**

Health system responsiveness measures (HSR) the non-health aspect of care relating to the environment and the way healthcare is provided to clients. The study measured the HSR performance and correlates of HIV/AIDS treatment and care services in the Wolaita Zone of Ethiopia.

**Methods:**

A cross-sectional survey across seven responsiveness domains (*attention*, *autonomy*, *amenities of care*, *choice*, *communication*, *confidentiality* and *respect*) was conducted on 492 people using pre-ART and ART care. The Likert scale categories were allocated percentages for analysis, being classified as unacceptable (Fail) and acceptable (Good and Very Good) performance.

**Results:**

Of the 452 (91.9%) participants, 205 (45.4%) and 247 (54.6%) were from health centers and a hospital respectively. 375 (83.0%) and 77 (17.0%) were on ART and pre-ART care respectively. A range of response classifications was reported for each domain, with Fail performance being higher for choice (48.4%), attention (45.5%) and autonomy (22.7%) domains. Communication (64.2%), amenities (61.4%), attention (51.4%) and confidentiality (50.1%) domains had higher scores in the ‘Good’ performance category. On the other hand, ‘only respect (54.0%) domain had higher score in the ‘Very Good’ performance category while attention (3.1%), amenities (4.7%) and choice (12.4%) domains had very low scores. Respect (5.1%), confidentiality (7.6%) and communication (14.7%) showed low proportion in the Fail performance. 10.4 and 6.9% of the responsiveness percent score (RPS) were in ‘Fail’ and Very Good categories respectively while the rest (82.7%) were in Good performance category. In the multivariate analysis, a unit increase in the perceived quality of care, satisfaction with the services and financial fairness scores respectively resulted in 0.27% (*p* < 0.001), 0.48% (*p* < 0.001) and 0.48% (*p* < 0.001) increase in the RPS. On the contrary, visiting traditional medicine practitioner before formal HIV care was associated with 2.1% decrease in the RPS.

**Conclusion:**

The health facilities performed low on the autonomy, choice, attention and amenities domains while the overall RPS masked the weaknesses and strengths and showed an overall good performance. The domain specific responsiveness scores are better ways of measuring responsiveness. Improving quality of care, client satisfaction and financial fairness will be important interventions to improve responsiveness performance.

**Electronic supplementary material:**

The online version of this article (doi:10.1186/s12913-017-2224-1) contains supplementary material, which is available to authorized users.

## Background

Clients’ satisfaction with the health services is a function of health systems, is impacted by perceived quality of care and the environments and how services are provided [1–5]. The awareness of client rights to respect and autonomy in medical care and decision making [5–7] has necessitated a responsive and effective health care system. In resource constrained settings, where many people are without the means to access private health care, the need to use clients’ perceptions to identify system weaknesses and its responsiveness to their needs is essential to improve the performance of government services [1, 2, 8]. Policy makers and resource allocators in developing countries need to understand and respond to their clients’ needs to ensure that the best possible quality of services are provided, given their lack of resources. The qualitative issues that affect client’s satisfaction such as the interactions between the clients and care providers, other facility staff and the health care environments need to be explored to understand their role in the provision of care [5, 8, 9].

Health systems are organizations and resources, their purpose being to provide health care to promote and ensure people’s wellbeing and protect them from disastrous cost of illness and care [8, 10]. Health care provision therefore involves many intertwined processes and interactions between care providers and clients in the course of service provision. The environments in which these happen affect the client’s perceptions as well as influence the people-centeredness of the healthcare provided [5]. Being consumers of the services with dissimilar biopsychosocial backgrounds, the clients present with different service demands and expectations that need to be attended to adequately and rightfully [5, 8]. Unmet expectations can be problematic, with dissatisfaction derailing client’s confidence in the care provider and the system [11–13]. As a result, incongruences in the clients’ expectations and performances need to be identified and monitored on an ongoing basis, and should be dealt carefully and as a priority.

Health system responsiveness (HSR) is a new concept that has evolved over the last decade and is drawing the attention of a few researchers [1, 2, 14–19]. It refers to clients universally legitimate expectations (as per the service standards and ethics principles), and measures the performance of the health systems in terms of the extent to which they provided services as a response to their client’s needs, as well as the environments in which they are served [1]. It encompasses eight domains: *autonomy* (involvement in medical decision making), *attention* (timeliness of care and due attention), *respect* (dignity and treatment with regard), *choice* (of care provider and units), *confidentiality* (keeping medical secrets and maintaining privacy), *communication* (interactions with service providers), *amenities of care* (convenience of facilities) and *access* to social support (family company, religious practices, etc.) [1, 2, 20].

HSR is one of the priority goals of health service development according to the World Health Organization (WHO) health system framework [21]. However, many health systems have not extended its application to the healthcare delivery level. The HSR domains are important indicators of the performance of health systems, how people-centered the healthcare is and to what extent the legitimate expectations of the clients are being met [5, 8, 11]. Studies show that the higher the HSR, the greater the chances of treatment successes, meeting the clients’ expectations and contentment with the services will be [10–13]. In medical conditions associated with chronic health impairments or compromised immune and recovery systems such as with HIV/AIDS, where lifelong treatments are prescribed and adherence is essential, people-centeredness of healthcare is invaluable for good treatment outcomes [5–7].

According to the Joint United Nations Program on HIV and AIDS (UNAIDS) global fact sheet, there were over 36.9 million people infected with HIV in 2014, with 41.0% being enrolled on antiretroviral therapy (ART) [22]. However, the report showed that 70% of all HIV infected people and 1.4 million new infections occurred in sub-Saharan Africa in that year. In addition, 67.0% of infected men and 57.0% of the women were not enrolled on ART, indicating the inequalities in access to HIV treatment. Although HIV prevalence was relatively low in Ethiopia in 2014 (1.3%) [23, 24], there were over 800,000 people infected with HIV, with wide regional variation within the country [24–26]. With ART scale-up through the Ethiopian public health system, the number of people enrolled has improved from a few thousand people in 2005 to 344, 344 in 2014 [26, 27]. Nevertheless, 57% of people living with HIV (PLHIV) [26] were not accessing ART in 2014. With the new WHO guideline to enroll all PLHIV on ART regardless of the CD4 count, the need for ART will increase markedly [28].

Despite the gains in ART scale-up and the reductions in morbidity and mortality to HIV/AIDS [27, 29, 30], there have been glaring gaps in implementing HIV/AIDS prevention and control programs in Ethiopia and globally [31–35]. Many studies reported that healthcare facilities lacked adequate financing, trained and motivated health workforce, and essential logistics. In addition, this situation was worsened by the poor quality of care, little or no regard for the socio-cultural factors that influence the perceptions and expectations of clients, and the inability of the health systems to create better care climates [33, 34, 36–38].

People on pre-ART (are PLHIIV who do not meet the admission criteria for ART) and ART care (are PLHIV who meet the ART admission criteria and are already on ART care), being stressed by their health conditions and the associated stigma [31, 39, 40] need healthcare systems that respond to their needs, meet their expectations and treat them with dignity [3, 5, 41] in order for them to adhere well to prescriptions and have better health outcome. However, to what extent these have been satisfied are not known, hindering improvements to health systems and disadvantaging clients from benefiting from better services. This study was therefore conducted to innovatively measure HSR performance and correlates in the context of HIV/AIDS treatment and care services (HATCS) in the Wolaita Zone of Ethiopia from November 2014 to March 2015.

## Methods

### Study area

The study was conducted in Wolaita Zone, which has 12 districts and three town administrations, with 324 kebeles (an equivalent of villages). It is located 163 km south west of Hawassa (the capital city of the Southern Nations, Nationalities and Peoples Region), and 330 km south west of the Ethiopian capital city of Addis Ababa. There were 1,866,400 inhabitants in 2014 and the zone was among the most densely populated areas in the country. Projections based on the 2007 Population and Housing Census of Ethiopia showed that 98.0% of the population were Christians and 96.8% spoke Wolaita Donna. The Census also indicated that approximately 46.1% were educated (literate) and 97.2% were employed, mainly in agriculture [42].

According to the zonal reports, the potential health coverage (percentage of population who have access to basic healthcare facilities) was over 95% in 2014 taking into account the health posts (community level frontline health facilities providing basic preventive and medical care) functioning in each village. In the same period, three hospitals, 63 health centers, 333 health posts, and several private clinics and drug venders operated in the zone. A total of 16 795 were estimated to be infected with HIV in 2014 in the zone (an assumption based on the regional 0.9% HIV prevalence rate as per the EDHS 2011 survey findings) [24, 25]. This study was part of a bigger study by BY and BPN and the information about the study area and sampling of participants has been published elsewhere [43, 44]. The Zone was chosen due to the reported challenges on utilization of HATCS, accessibility of health facilities and availability of adequate number of people infected with HIV using HATCS for the study.

### Sample size, sampling and participant selection

The study population were all people infected with HIV in Wolaita Zone while the sample population were those obtaining HATCS (pre-ART and ART care) at the outpatient HIV care units of one zonal (teaching) hospital and five health centers The selected facilities served 74.6% (2262 out of 3038) of all people infected with HIV on ART care in the zone in 2014. The sample size for ART clients was determined by using the one-sample population proportion formula [45] with a 50.0% probability of the responsiveness of HATCS (there were no local data available on the subject), an α – error of 5%, an 80% power and a 95% confidence interval [45]. The calculation indicated a sample size of 385 with a 10% non-response contingency being added, making the total sample size required 424.

The number of ART clients selected from each of the six facility was determined based on the *probability proportional to size*. In each facility, using the ART client registration number, the principal investigator (PI) randomly selected the potential participants from six health facilities, each of whom was assigned a unique code and forwarded to the ART unit nurses stationed at the facility. When the identified clients appeared in the health facility for their routine medical care such as checkups and drug refills, the nurses provided them with information about the proposed study and asked if they would like to participate. When the clients agreed, the nurses obtained written consent and connected them with data collectors. Regarding the pre-ART clients, all clients registered for the pre-ART service were invited to participate in the study due to the small number of clients available in this category of HIV care. Similar consenting procedures were followed for people on pre-ART care. The entry criteria for the study were age ≥18 years, attending an outpatient care, permanent resident in the study area, outpatients (who were not in pain) and able to give consent. All clients who did not meet these criteria were excluded. Participation was voluntary and informed consent was obtained from all study participants.

### Data collection instrument and variables

A closed-ended interview questionnaire was adapted from the health systems responsiveness questionnaires used in the WHO multi-country studies [14, 46, 47]: the Health Care Climate Questionnaire (HCCQ) short form [48], the Patient Health Questionnaire (PHQ 9), the SERVQUAL [49, 50] and Lei and Jolibert (2012) scales [51]. The responsiveness questions in the adapted tool were grouped under seven domains with 44 questions that had ordinal response categories. The HCCQ short form’s six questions were adapted to qualify the autonomy domain of the WHO’s responsiveness questionnaire. The study did not include the ‘access to social support’ domain as it is not applicable for outpatient care [2].

Each question was rated with four, five or seven point Likert scale options (responses coded 1–4, 1–5 and 1–7 respectively) depending on the type of questions presented i.e. from ‘never’ to ‘always’, ‘very bad’ to ‘very good’ or ‘strongly disagree’ to ‘strongly agree.’ The 44 questions were divided among the seven domains of responsiveness: prompt attention (7), respect (7), and communication (7), quality of basic amenities (11), confidentiality (3), choice (3) and autonomy (6). The internal consistency of the overall responsiveness scale (44 items) as measured with the Cronbach’s alpha was 0.880. The domains of responsiveness had the Cronbach’s alpha between 0.760 and 0.940, indicating good internal consistency.

Socio-demographic data were obtained from participants to enable evaluation of the results against the clients’ backgrounds and health status data such as HIV clinical stage, CD4 count and type of HIV care were obtained from medical records. In the instrument, geospatial factors (distance, difficulty of the topography and transportation accessibility), relative financial fairness, time spent to obtain services, satisfaction with the services. The perceived quality of care questions were included to assist in exploring factors associated with health system responsiveness. The questionnaire was prepared in English and translated into Wolaita Dona and Amharic for use after being piloted. Using 20 ART clients who were not included in the final data collection, the instrument was piloted and checked for any problems in interviewing and administration. For instance, questions about the “access to social support” domain of the responsiveness were dropped and some questions were rephrased.

The dependent variable of the study was responsiveness of HATCS that was computed as responsiveness percent score (RPS) from the total responsiveness score (TRS) as detailed in the data analysis section. The independent variables included age, sex, educational status, years spent on education, perceived own health, religion, marital status, income and employment status. In addition, CD4 count, PHQ-9 score, HIV clinical stage, co-infection with TB (Yes/No), HIV sero-status disclosure (Yes/No), distance from health facility, out of pocket expenses (Yes/No), perceived financial fairness score, perceived quality of care score, satisfaction with care score and time spent on obtaining care were used as independent variables of the study.

The perceived financial fairness score was computed from eight questions about the relative worth of care for the clients against the amount of money spent. The clients rated the questions from five which were coded 1–5 i.e. ‘very bad’ to ‘very good’ respectively. Similarly, the total perceived quality of care score was computed by adding the responses to 13 questions of the clients’ perceptions about the services, professionalism of the care provider, regard for the patient values and interests in the service, delivery of care on the right time and the extent to which the clients’ expectation about the quality were met in each service outlet. Similarly, the satisfaction with care was computed by adding the responses to six questions about how well the clients were satisfied with the services offered in each of the service outlets. The clients were asked to rate their satisfaction with the service on a five-point Likers scale (coded 1–5) from ‘very dissatisfied’ to ‘very satisfied.’ The internal consistencies of financial fairness, perceived quality of care and satisfaction with care scales were conducted i.e. they had 0.810, 0.880 and 0.830 Cronbach’s alpha respectively which were well above the cut-off of 0.700 required.

Nine local data collectors were employed who were fluent in both Wolaita Dona and Amharic and had at least a Diploma in Nursing or BSc in Public Health. None of them were staffs nor were affiliated to the facilities they were assigned. Ten nurses and focal persons of the HIV care units of the health facilities (in which the study was conducted) were oriented about the study.

### Data analysis

The data were analyzed using STATA 13.1 (StataCorp, Texas, USA) and consisted of two stages, the descriptive analysis of the responsiveness performances and inferential statistics to determine factors associated with the total responsiveness of the health systems of HATCS. The descriptive analysis converting the results of all the respondents Likert scale replies into percentages was complicated by the domains having 4, 5 and 7 response options and resulted in setting different cut-offs for the responsiveness performance categories. The Likert scale rating for each domain was matched with the responsiveness performance categories as ‘unacceptable’ (Fail) and ‘acceptable’ (Good and Very Good). For instance, the corresponding code for ‘neutral’ response for autonomy domain was four that was multiplied by six (the number of questions in the domain) that produced a cut-off score of 24 for ‘Fail’ HSR performance. This cut-off score was multiplied by 100.0% and divided by 42 (the maximum possible responsiveness score for the domain) that 57.1 being the % cut-off for Fail performance category for autonomy. The % cut-off for all responsiveness domains and the responsiveness performances were computed similarly (see Additional file 1). Therefore, an increase in the RPS or TRS is considered as better/positive performance of the healthcare while a decrease is weak/negative performance or undesired one. Similarly, increases in satisfaction, financial fairness and perceived quality of care scores are better/positive performance.

First, all respondents scores for the seven domains were converted to percent i.e. the scores divided by the maximum possible scores multiplied by 100%. Second, the total possible scores for each of the response categories such as ‘very bad’, ‘bad’, etc. for each of the domains were converted to percent (after dividing by the maximum responsiveness score for the domain multiplied by 100%). Third, these percent scores for the response categories were used as cut-off points to group the responsiveness performances of the healthcare.

The RPS for the domains that were between the minimum and including the cut-off for the acceptable performance were regarded as ‘Fail’ (Table 1). Those percent performances above the cut-off for good and including the cut-off for ‘very good’ were categorized as ‘Good’. Finally, those performances above the cut-off for very good were regarded as ‘Very Good’. The minimum RPS for each domain was calculated using the minimum responsiveness scores such as autonomy, 6; attention, 7; communication, 7; amenities, 11; choice, 3; confidentiality, 3; and respect, 7. The total responsiveness score was computed for each of the 44 questions that weighted on the maximum possible score (219) and converted to the responsiveness percentage scores (RPS) i.e. TRS of the individual clients observations were multiplied by 100.0% and divided by 219 (see Additional file 1).

**Table 1:** Classification Responsiveness Performance

Responsiveness Domain	Minimum RPS^a^ (%)	RPS^a^ Categories		
		Unacceptable	Acceptable	
		Fail (%)	Good (%)	Very Good (%)
1. Autonomy	14.4	14.4–57.1	57.2–75.0	75.1–100.0
2. Attention	20.0	20.0–60.0	60.1–80.0	80.1–100.0
3. Communication	20.0	20.0–60.0	60.1–80.0	80.1–100.0
4. Amenities	20.0	20.0–60.0	60.1–80.0	80.1–100.0
5. Choice	25.0	25.0–50.0	50.1–75.0	75.1–100.0
6. Confidentiality	25.0	25.0–50.0	50.1–75.0	75.1–100.0
7. Respect	25.0	25.0–50.0	50.1–75.0	75.1–100.0
Total Responsiveness	20.1	20.1–58.9	59.0–79.9	80.0–100.0

^a^ Responsiveness percent score

The univariate and bivariate analyses were performed to describe the frequency, distribution and binary relationships. The data were visually inspected using the graphs for normality, linearity of relationships and the presence of outliers in the dependent variable and residuals. The outliers observed in the univariate analysis and during the post estimation in the multivariate analysis were removed to ensure the fitness of the model, increase the percentage of variations explained by the variables entered to the multivariate model and to have dependable estimations. To check for the fulfilment of the first basic assumption i.e. normality of the dependent variable, Shapiro-Wilk W test for normal data was conducted and showed *p* ≥ 0.050 (*p* = 0.259).

J Stock and MW Watson [52] suggested the use of *robust* estimation methods that relaxes and accounts smoothly for heteroscedasticity in multiple linear regressions. Heteroscedasticity, the equality of the variances in the linear regression model, was numerically tested with the Breusch-Pagan/Cook-Weisberg test showed the *p*-value of 0.602 that satisfied the assumption. The model specification was checked with the post-estimation methods and proved that the assumption was satisfied in the model. In addition, the Ramsey RESET test showed there were no omitted correlated variables that significantly affected the model (*p*-value = 0.550). The residuals were tested for normality and showed the *p*-value of 0.099, the assumption being satisfied.

Visual inspection of the model showed there were no patterns between the residuals and fitted values, confirming that the distributions occurred randomly. Similarly, multicollinearity among the independent variables was checked with the variable inflation factor (*vif*) estimation after the regression analysis, which showed a mean value of 1.430 with none of the individual estimations for the factors exceeding 2.010. How much each observation influenced the overall model was checked with the Cook’s distance, which showed a ≤ 0.040 value i.e. comfortably below the conservative cut-off point of 0.500. Finally, three-way full factorial analyses were conducted between the suspected factors, e.g. the perceived quality of care, satisfaction with care and financial fairness scores, with none of the interactions being found to be statistically significant, and only the main effects being considered in further analysis.

### Ethical considerations

Ethical clearance was obtained from the Biomedical Research Ethics Committee (BREC) of the University of KwaZulu–Natal (South Africa) and Wolaita Soddo University (Ethiopia). Permission was obtained from the health facilities and the district health offices to access medical records and to contact the selected clients who were required to sign informed consent to participate.

## Results

### The socio-demographics and health characteristics

Out of 492 participants (411 on ART and 81 on pre-ART) interviewed, the observations of 452 (91.9%) were included in the analysis after removing 11 (2.2%) with missing values and 29 (5.9%) that were outliers. The response rate was 96.9% for people on ART as eight patients could not be reached and three were either seriously ill or inpatients. On the other hand, response rate was not computed for people on pre-ART as all coming for the service were invited to participate. Of the 452, 205 (45.4%) were from the health centers while 247 (54.6%) were from the hospital. Of them, 375 (83.0%) were on ART care and 77 (17.0%) were on pre-ART care. Regarding the HIV/AIDS clinical status of the participants, 330 (73.0%) were at Stage 1, 99 (21.9%) at Stage 2 and 23 (5.1%) at Stage 3, as indicated on their medical records. Their CD4 counts (last count in the preceding 6 months) were 194 (44.5%) of <500/cm3 and 242 (55.5%) for ≥500/cm3. Regarding their socio-demographics, 185 (40.9%) were males and 267 (59.1%) were females, while 305 (67.5%) and 147 (32.5%) were urban and rural residents respectively (Table 2).

**Table 2:** Participants’ Socio-demographic characteristics

Variable	Response Category	Number	Percent
Age in years (*n* = 461)	Range	18–71	
	Mean±SD^d^	34.8 ± 8.8	
Education	Not educated	80	17.7
	Grade 1–4	67	14.7
	Grade 5–8	150	33.2
	High school	101	22.4
	Vocational School	14	3.1
	College/university	40	8.9
Current Marital Status	Married	229	51.1
	Not married^a^	219	48.9
Employment	Employed	323	72.1
	Not employed	125	27.9
Monthly Income (ETB) ^c^	Range	100–4000	
	Average	584.5 ± 480.93	
Religion	Orthodox Christian	173	40.6
	Protestant Christian	253	59.4

^a^ Single, divorced, separated and widowed; ^b^ unemployed, student or retired; ^c^ Ethiopian Birr (currency); ^d^ standard deviation

### Responsiveness of the healthcare

The minimum and maximum of the TRS were 101 and 194 respectively out of a possible 219, with the mean of 153.1 ± 16.7. The total RPS were from 49.3 to 86.2%, and the 25th, 50th and 75th percentiles of the HATCS TRS were 63.1, 68.0 and 73.1% respectively, with the mean score of 68.3 ± 7.4%. The lowest mean score was observed in the *choice* domain (57.7%) while the highest mean percent score (81.7%) was in the *confidentiality* domain (Table 3).

**Table 3:** Responsiveness Performances of HATCS (*n* = 452)

Responsiveness Domains	Responsiveness % Score, Mean [95% CI]	Min–Max % score	Unacceptable (%)	Acceptable (%)		
			Fail	Good	Very Good	Total
Autonomy	71.2 [69.6–72.9]	28.6–100.0	22.7	43.1	34.2	69.3
Attention	64.6 [63.8–65.5]	31.4–100.0	45.5	51.4	3.1	54.5
Communication	76.1 [74.9–77.4]	37.1–100.0	14.8	64.2	21.1	85.3
Amenities	65.2 [64.1–66.2]	38.2–100.0	33.9	61.4	4.7	66.1
Choice	57.7 [55.4–59.8]	25.0–100.0	48.4	39.2	12.4	51.6
Confidentiality	81.7 [80.1–83.2]	25.0–100.0	7.6	50.1	42.3	92.4
Respect	77.8 [76.6–78.9]	28.6–100.0	5.1	40.9	54.0	94.9
Total Responsiveness	68.3 [67.6–68.9]	49.3–86.2	10.4	82.7	6.9	89.6

*CI* confidence interval

The responsiveness domains and RPS were computed using the cut-off points, as indicated in Table 3. A range of response classifications was reported for each domain, with Fail responsiveness performance being higher for choice (48.4%), attention (45.5%) and autonomy (22.7%) domains. Good responsiveness performance was higher for communication (64.2%), amenities (61.4%), attention (51.4%) and confidentiality (50.1%). Very Good performance was high for respect (54.0%). Very Good responsiveness performance was very low for attention (3.1%), amenities (4.7%) and choice (12.4%) domains. Respect (5.1%), confidentiality (7.6%) and communication (14.7%) showed low proportion in the Fail responsiveness performance category. The Fail, Good and Very Good responsiveness performance categories for the TRS were 10.4, 82.7 and 6.9% respectively. The percentage of clients who provided Acceptable rating for each of the responsiveness domains: respect (94.9%), confidentiality (92.4%), communication (85.3%), autonomy (69.3%), amenities (66.1%), attention (54.5%) and choice (51.6%).

### Factors associated with the responsiveness of HATCS

In the bivariate analysis, educational status, religious affiliation, family income, participant residence, marital status, distance from the health facility and HIV clinical stage did not show any statistically significant association with the RPS. However, the mean RPS of HATCS were significantly different for the type of health facility, employment status, out of pocket expense, perceived own health and visiting traditional medicine practitioner before starting on the pre-ART or ART care (*p* < 0.050). In addition, the perceived quality of care, financial fairness and satisfaction with care scores had statistically significant positive associations with the RPS (Table 4 column 3). Variable such as socio-demographics, PHQ 9, CD4 count, HIV disclosure (Yes/No), geospatial factors and HIV/AIDS clinical stage did not show statistically significant association with RPS and were not considered multivariate analysis.

**Table 4:** Factors Associated with the Responsiveness of HIV/AIDS Treatment and Care Services (*n* = 452)

Correlated Variables	Response Categories	Coefficients (Bivariate Linear Regression Analysis)	Coefficients (Multivariate Linear Regression Analysis)
Type of Care Registered	ART	−2.62 [(−4.44)–(−0.81)]^***^	
	Pre-ART	Ref (0.00)	
Type of Health Facility	Health Center	4.29 [2.98–5.62]^***^	
	Hospital	Ref (0.00)	
Employment status	Employed	Ref (0.00)	Ref (0.00)
	Not-employed	2.11 [0.59–3.64]^**^	0.41 [(−0.58)–1.41]
Out of pocket expense	Yes	−2.91 [(−4.59)–(−1.22)]^**^	−0.68 [(−0.46)–1.83]
	No	Ref (0.00)	Ref (0.00)
Perceived health	Good	Ref (0.0)	Ref (0.0)
	Not Good	−3.12 [(−4.92)–(−1.32)]^***^	−0.01 [(−0.99)–1.16]
Visited traditional Medicine provider	Yes	−4.63 [(−5.98)–(−3.29)]^***^	−2.12 [(−3.05)–(−1.18)]^***^
	No	Ref (0.0)	Ref (0.0)
Scores	Perceived quality of care	0.50 [0.45–0.56]^***^	0.27 [0.20–0.34]^***^
	Satisfaction with care	1.56 [1.37–1.75]^***^	0.48 [0.27–0.70]^***^
	Perceived financial fairness	0.89 [0.79–0.99]^***^	0.48 [0.38–0.58]^***^

^*^ *p* < 0.05; ^**^ *p* < 0.01; ^***^ *p* < 0.001

In the bivariate analysis (Table 4 column 3), the clients of the health centers had 4.3% more RPS than the hospital clients (*p* < 0.010). Similarly, a unit increase in the perceived quality of care, satisfaction with services and perceived financial scores resulted in 0.5, 1.6 and 0.9% increase in the responsiveness score respectively (*p* < 0.001). The unemployed clients had 2.1% more responsiveness scores than their employed counterparts (*P* < 0.050) when other associated factors were not considered.

Similarly, in the bivariate analysis, the people on ART care had 2.6% less RPS than the people on pre-ART care (*p* < 0.001), and the RPS decreased by 2.9% when clients spent money out of pocket than when they did not (*p* < 0.010). In addition, when the clients felt their health was not good (when they felt they were sick), the responsiveness score decreased by 3.7% than when they did not, and when they had visited traditional medicine practitioner before the formal healthcare, the RPS decreased by 3.1% (*p* < 0.001).

The variables significantly associated with the RPS were included in the multivariate analysis (Table 4 column 4) and analyzed using the *regress* model. The model fitting in the multiple regression showed *p* < 0.001 and the majority of variations (62.0%) in the model were explained by the independent variables included in the model. In addition, when the number of independent variables were accounted for and the multiple regression model was corrected, 61.0% of the variations in the model were explained by the variables. When adjusted, the coefficients of determination of the type of health facility and type of care registered reversed or showed not significant associations, and the factors were excluded. The removal of these factors did not affect the model fitting and associated statistics such as the R-squared, coefficients and *p*-values, due to the small effect they had on the variations in the model.

The multiple regression analysis (Table 4 column 4 showed that the perceived quality of care, satisfaction with the services and perceived financial fairness scores were positively associated with the RPS when other factors were held constant (*p* < 0.001). A unit increase in the perceived quality of care was likely to result in 0.3% increase in the RPS when other factors were kept constant (*p* < 0.001). Similarly, a unit increase in the satisfaction with the services score was likely to result in 0.5% increase in the RPS (*p* < 0.001). Accordingly, the participants with higher financial fairness score were more likely to have higher RPS i.e. a unit increase in the financial fairness score was likely to result in 0.5% increase in the RPS if other factors were kept constant (*p* < 0.001).

However, visiting traditional medicine practitioner before the formal HATCS was associated with 2.1% decrease in the RPS when other factors were kept constant (*p* < 0.001). Other factors such as out of pocket expenses, employment status and perceived own health status became statistically not significant when adjusted for other factors (*p* > 0.05).

## Discussions

As an emerging area of research, recent studies on health system responsiveness have focused on validating study instruments and its domains [53–55]. A few studies were conducted to assess the HSR on specific health conditions, such as the patients with heart failures [56], hospitalized patients [17], health insurance users [57] and women who gave birth at a hospital [15], and used different methods and showed varying results. The initial studies conducted by the WHO and other experts focused on the inter-country comparison of the overall performance of the HSR and developing frameworks and testing their applicability [1, 2, 20, 58, 59]. As a result, the discussions in the following paragraphs are limited to the information available on HSR for comparison.

### The socio-demographics and health characteristics

The results of the study showed the socio-demographic characteristics and the health status of the people on ART and pre-ART were not significantly associated with the RPS. This might indicate the clients in different socio-demographic categories rated the healthcare responsiveness equally or if differences were observed, they were due to errors, chances, confounding or occurred randomly (*p* ≥ 0.050). However, a study in Nigeria showed type of facility, gender, educational status, marital status and income were significantly associated with health system responsiveness [57]. The differences in the results might indicate the approaches used in the inquiry and analysis of data. For instance, the Nigeria study participants were insurance enrollees regardless of what health conditions made them visit the health facilities while the participants of our study were only either people on ART or pre-ART care. In addition, the Nigeria study used only six domains of responsiveness while this study used seven domains of responsiveness. Cultural and contextual differences might have played an important role in perceptions and expectations of health system responsiveness that could explain the differences observed. More studies are required to understand how the socio-demographic and health characteristics of the individual clients (of different health services) correlated with the responsiveness of health systems.

### Responsiveness of the healthcare

The responsiveness performances of the healthcare varied widely across the domains, a large proportions of the rating of prompt attention, choice and amenities falling in the ‘fail’ performance category. Low proportions of ratings in the responsiveness domains were observed in the ‘very good’ category i.e. for the prompt attention, amenities of care and choice, with only one getting slightly over 50.0%. A study in Iran showed similar findings, such as autonomy and choice of provider being the least performing domains of responsiveness in hospitals [18], while another study in Iran showed choice and prompt attentions domains being the least performing domains for outpatients of chronic heart failure condition in a hospital [56].

The results showed gaps in healthcare facilities responsiveness performances, indicating that they failed to meet the expectations of clients regarding how they should be treated and the convenience of the environments in which they were treated (non-health aspects of the medical care). A qualitative study conducted in the study area showed that the ways clients treated in the health facilities varied across health facilities and care providers and impacted the perceptions of clients towards responsiveness [60]. However, the findings also showed that all of the responsiveness domains had substantial proportions of their performance falling in the ‘good’ category, which meant that some expectations regarding the responsiveness domains were met. With targeted and mindful interventions such as training of healthcare workers and on-site mentoring and technical assistance, these ‘good’ performances could be opportunities to be transformed into ‘very good’ performances. The results generally indicated the need to improve the responsiveness performances.

The total responsiveness performance figures showed the average masked the weak and strong responsiveness performances. As a result, total responsiveness performance could be useful when the health facilities target improving their performances in all domains of responsiveness or when the intention is to compare the overall responsiveness performance among healthcare facilities. However, when the purpose is to better understand on which aspect the healthcare was performing well or bad, the domains-based responsiveness evaluation might be necessary. Therefore, more studies need to be conducted to investigate if the analysis methods used in this study could be replicated elsewhere or in other health conditions such as the clinical management of childhood illnesses, malaria, obstetric problems, etc.

The absence of standardized references to measure healthcare responsiveness performance was an obstacle in rating of the participant’s responses which could impact on identifying and planning interventions to improve the responsiveness performances of the HATCS. This study therefore developed a tool which enables healthcare facilities to evaluate their performances without the need to look for national or regional references. Matching the responses to each of the questions in the domains of responsiveness with the acceptable and unacceptable performance categories provides internal reference (cut-off) scores. Second, these reference scores when converted to percent (divided by maximum score for the domain and multiplied by 100.0%) show how the health facilities are performing and satisfying the clients. However, this approach was an early attempt and more studies should be conducted to improve the tool’s wider application.

### Factors associated with the responsiveness of HATCS

The results of the study showed that the perceived quality of care positively influenced the responsiveness of HATCS. The quality of care as a critical component of every healthcare [4, 5, 61] was found to be the correlate of responsiveness. Acknowledging the tendency of bidirectional determinism between the two variables, it is important for the healthcare facilities to improve the providers’ professionalism (expertise), ability to understand the psychological and physical conditions of the client and provide appropriate treatment for the clients. In addition, the health care system and care providers in particular need to show regard for the interests of the clients and provide care as mandated to score better in the perceived quality of care.

Satisfaction with services was a significant correlate of the responsiveness performances of the HATCS. Similar to the perceived quality of care, the satisfaction with care is linked to the experiences and expectations of the clients and affects their evaluation of the healthcare performance [5, 13, 62]. Due to its strong attachment to the psychological state of the clients [5, 12, 63], providing comprehensive and quality services will be necessary to improve the responsiveness of healthcare. For instance, further study by the authors of this study on perceived quality of care and satisfaction with services of HATCS showed that HSR was positively correlated with them [43, 44] that improving one will result in betterment of the other factor. This may be achieved through staff training, availing service standards and supervision.

Although most HIV/AIDS treatment services were offered free of charges (including ART) in the study area, there were instances the health facilities being short of drugs and supplies (for the treatment of opportunistic infections) making it necessary for patients to purchase their medication from private pharmacies. This is an additional expense to their travel costs to reach the facility and is a challenge for the poor. Despite this fact, the perceived financial fairness and not the out of pocket expense was found to be a significant correlate of the responsiveness of the HATCS. It appeared that the relative worth of the expenses (financial fairness) against the quality of care and met expectations influenced the rating of the responsiveness of HATCS. Studies elsewhere [5, 64, 65] also indicated the importance of reducing cost and increasing financial fairness of the healthcare to enable clients to access services and improve its perceived quality. Other studies also reported that better quality of care and relative worth of the care compensated the discontent with increased expenses [64, 66–68]. By ensuring the financial fairness and improving the quality of care, the health facilities could be rated more responsive.

Clients who first visited traditional medicine practitioner negatively impacted their rating of the responsiveness of HATCS. This preference for first consulting the traditional medical practitioners suggests that they address the psychological needs of the clients in the way the clients want [69]. In addition, the traditional medicine practitioners generally usually live in the same community by the clients.

Studies reported the existence of poor integration of the traditional medicine with the health facilities [69, 70], resulting in competition and rivalry rather than cooperation. However, studies also have indicated that conjoint service delivery with the traditional practitioners improved the outcomes of treatments [2]. Further studies are needed to understand the reasons for the negative relationships of the clients who first visited the traditional medicine practitioner. The study findings suggest the integration of the formal healthcare with traditional medicine might be important to harmonize the performances of HATCS [71].

## Strengths and limitations of the study

Regarding the limitations of the study, as this was a cross-sectional study, temporality of the associations between the factors associated with HSR could not be established. The study participants were asked to rate health facilities performance based on their experiences that mainly depends on their perceptions which could not be measured objectively. HSR is a newer concept that literature is short for discussion. Most of the participants were people on ART that cautions should be taken while generalizing the findings. The findings of the study might be substantiated with qualitative studies. The strengths of the study were that it innovatively measured an often neglected aspect of healthcare and shined light on how to do so. A relatively large sample size was used that the results could be considered representative of HATCS in the study area.

## Conclusions and recommendations

In resource constrained countries, where providing health services are constrained by limited resources, understanding clients’ needs can assist in identifying where these resources need to be spent. It also indicates where improvements in staff training and service environments can occur to ensure that the services are provided with respect and the clients’ legitimate expectations are met. This includes people infected with HIV need robust and comprehensive HATCS where their psychosocial and health needs can be satisfied to ensure and encourage to adhere to the lifelong treatments and live positively with the disease.

Based on the findings, strengths and limitations of the study, the following conclusions and recommendations are made. The responsiveness performance of the HATCS varied across the domains i.e. substantial proportions of the ratings of prompt attention, choice and amenities falling in the ‘fail’ (unacceptable) performance category. On the other end, these three domains had the lowest proportions in the very good responsiveness performance category indicating the need for improvement.

The larger proportion of responsiveness performance in the good category presents an opportunity for the healthcare to transform their performance to very good. This could be achieved if the health care system considers the correlates of the responsiveness of HATCS such as perceived quality of care, satisfaction with the services, financial fairness and integration of the formal healthcare with the traditional medicine. The methods and tools used to measure health system responsiveness can be useful to evaluate the performance of HIV care in resource limited settings such as Ethiopia. Conducting similar evaluations in other settings and health services will enable the tools and methods used to be refined and advanced.

Further studies may be required to confirm to what extent these correlates contributed to other health conditions and contexts. This could include investigating how the clients’ backgrounds and their health status were related to the correlates of the responsiveness of healthcare, specifically the HATCS. In addition, how and why the traditional medicine plays in the clients’ evaluations needs further studies to better understand and plan for future improvements.

## Additional file

Additional file 1: Categorization of Participant Responses to Responsiveness Performances. Describes about the categorization of responsiveness performances of each of the respondents and shows the cut-off points for each of the responsiveness domains using the response categories for each of the questions in the domains. (PDF 110 kb)

## Abbreviations

ART: Anti-retroviral therapy
BREC: Biomedical Research Ethics Committee of the University of KwaZulu–Natal (South Africa)
HATCS: HIV/AIDS treatment and care services
HCCQ: Health Care Climate Questionnaire
HSR: Health system responsiveness
PHQ 9: patient health questionnaire (9 item)
PLHIV: People living with HIV
Pre-ART: Pre-antiretroviral therapy
RPS: Responsiveness percent score
TB: Tuberculosis
TRS: Total responsiveness score
UNAIDS: Joint United Nations Program on HIV and AIDS
WHO: World Health Organization
