# Supplementary material for: Measuring health system responsiveness at facility level in Ethiopia: performance, correlates and implications
Source: BMC Health Serv Res. 2017 Apr 11;17:263. doi: 10.1186/s12913-017-2224-1 (PMC5387185; doi:10.1186/s12913-017-2224-1)
Supplement: Additional file 1: — Categorization of Participant Responses to Responsiveness Performances. Describes about the categorization of responsiveness performances of each of the respondents and shows the cut-off points for each of the responsiveness domains using the response categories for each of the questions in the domains. (PDF 110 kb) [file 12913_2017_2224_MOESM1_ESM.pdf]

Additional file 1 Categorization of Participant Responses to Responsiveness Performances

Responsiveness Doman (no. of questions)	*Responses Categorized Fail*	*Minimum % score (No. of questions *100/D)*	*Cut-off scores for fail (B)*	*% cut-off Fail (B*100%/D)*	*Responses Categorized Good*	*Cut-off scores for Good (C)*	*% cut-off for Good (C*100%/D)*	*Responses Categorized Very Good*	*Maximum Score (D)*	*Maximum % score (D*100%/D)*
Autonomy (6)	Extremely disagree, strongly disagree, disagree, neutral	14.4	24	57.1	Agree, strongly agree	36	75.0	Extremely agree	42	100.0
Attention (7)	Very long, long	20.0	21	60.0	Usually	28	80.0	Always	35	100.0
Communication (7)	Very bad, bad, neutral	20.0	21	60.0	Good	28	80.0	Very Good	35	100.0
Amenities (11)	Very bad, bad, neutral	20.0	33	60.0	Good	44	80.0	Very Good	55	100.0
Choice (3)	Never, only sometimes	25.0	6	50.0	Usually	9	75.0	Always	12	100.0
Confidentiality (3)	Never, only sometimes	25.0	6	50.0	Usually	9	75.0	Always	12	100.0
Respect (7)	Never, only sometimes	25.0	14	50.0	Usually	21	75.0	Always	28	100.0
Total Responsiveness (44)	Extremely disagree, strongly disagree, disagree, neutral, very long, long, very bad, bad, never, only sometimes	20.1	129	58.9	Agree, strongly agree, usually, good	175	79.9	extremely agree, always, very good	219	100.0

Computations	*Responsiveness Domain (No. of questi ions)*							*Total Responsiveness (44)*
	Autonomy (6)	*Attention (7)*	*Communication (7)*	*Amenities (11)*	*Choice (3)*	*Confidentiality (3)*	*Respect (7)*	
Responses Categorized 'Fail'	Extremely disagree, strongly disagree, disagree, neutral	Very long, long	Very bad, bad, neutral	Very bad, bad, neutral	Never, only sometimes	Never, only sometimes	Never, only sometimes	Extremely disagree, strongly disagree, disagree, neutral, very long, long, very bad, bad, never, only sometimes
*Minimum % score (No. of questions *100/D)*	*14.4*	20.0	20.0	20.0	25.0	25.0	25.0	20.1
Cut-off scores for 'Fail' (B)	24	21	21	33	6	6	14	129
*% cut-off 'Fail' (B*100%/D)*	57.1	60.0	60.0	60.0	50.0	50.0	50.0	58.9
Responses Categorized 'Good'	Agree, strongly agree	Usually	Good	Good	Usually	Usually	Usually	Agree, strongly agree, usually, good
Cut-off scores for 'Good' (C)	36	28	28	44	9	9	21	175
*% cut-off for 'Good' (C*100%/D)*	75.0	80.0	80.0	80.0	75.0	75.0	75.0	79.9
Responses Categorized 'Very Good'	Extremely agree	Always	Very Good	Very Good	Always	Always	Always	Extremely agree, always, very good
Maximum Score (D)	*42*	35	35	55	12	12	28	219
*Maximum % score (D*100%/D)*	100.0	100.0	100.0	100.0	100.0	100.0	100.0	100.0
